# Searching for Novel Molecular Prognostic Markers in Colorectal Cancer—The Tumor Suppressor Proteins p53 and PTEN

**DOI:** 10.3390/biomedicines14071453

**Published:** 2026-06-26

**Authors:** Bartosz W. Bichalski, Magda Bichalska-Lach, Michał Nycz, Mariusz Kryj, Mirosław Śnietura, Dariusz Waniczek

**Affiliations:** 1 Department of Surgical Nursing and Surgical Propaedeutics, Department of General, Colorectal, and Multiple Trauma Surgery, Faculty of Health Sciences, Silesian Medical University, 40-055 Katowice, Poland; mbichalska@sum.edu.pl; 2 Department and Clinical Ward of Surgical Oncology, Faculty of Medical Sciences in Zabrze, Medical University of Silesia, 40-055 Katowice, Poland; michalt.nycz@gmail.com (M.N.); mhkryj@op.pl (M.K.); 3 Department of Pathomorphology and Molecular Diagnostics, Faculty of Medical Sciences in Katowice, Medical University of Silesia, 40-055 Katowice, Poland; miroslaw.snietura@sum.edu.pl

**Keywords:** colorectal cancer, adenocarcinoma, p53, PTEN, molecular biomarkers, survival analysis

## Abstract

**Background:** Colorectal cancer (CRC) remains a leading cause of cancer-related morbidity and mortality worldwide. While established molecular biomarkers such as microsatellite instability (MSI), KRAS, and BRAF are routinely used in clinical practice, the prognostic relevance of tumor suppressor proteins p53 and PTEN remains incompletely defined, particularly when assessed using immunohistochemistry. **Objective:** The primary aim of this study was to evaluate the prognostic significance of p53 expression and PTEN deficiency in colorectal adenocarcinoma. Secondary aims included assessment of their association with clinicopathological characteristics. **Methods:** This retrospective cohort study included 103 consecutive patients who underwent surgical resection for colorectal adenocarcinoma. Immunohistochemical analysis of formalin-fixed paraffin-embedded (FFPE) tumor samples was performed to assess aberrant p53 expression and PTEN deficiency. Associations with clinicopathological variables were evaluated, and overall survival was analyzed using Kaplan–Meier curves and Cox proportional hazards regression models. **Results:** Aberrant p53 expression and PTEN deficiency were both associated with shorter overall survival in univariate analyses. Patients with concurrent aberrant p53 expression and PTEN deficiency demonstrated the poorest survival outcomes. However, in multivariate Cox regression analysis, only nodal status and age remained independent predictors of overall survival, while p53 and PTEN did not retain independent prognostic significance after adjustment for clinicopathological variables. **Conclusions:** Aberrant p53 expression and PTEN deficiency are associated with reduced overall survival in colorectal cancer; however, their prognostic impact appears secondary to established clinicopathological factors. The combined presence of these alterations may identify a biologically aggressive subgroup of patients with particularly unfavorable outcomes, although this observation should be considered exploratory. Further validation in larger, independent cohorts is required.

## 1. Introduction

Colorectal cancer (CRC) is one of the most common malignancies worldwide and remains a major cause of cancer-related mortality [1]. According to Global Cancer Statistics 2022, CRC accounted for approximately 9.6% of all new cancer cases and 9.3% of cancer deaths globally, ranking as the third most frequently diagnosed cancer and the second leading cause of cancer-related death worldwide [2]. Despite advances in screening and treatment, the global burden of CRC continues to increase, particularly in low- and middle-income countries. Importantly, recent epidemiological data indicate a rising incidence of early-onset CRC among individuals younger than 50 years [3].

The prognosis of CRC is strongly stage-dependent. Five-year survival rates reach 94.7% for stage I, 88.4% for stage II, 74.3% for stage III, and 31.5% for stage IV disease [4]. Endoscopic screening, particularly colonoscopy, reduces CRC incidence and mortality; however, it is resource-intensive and adherence remains suboptimal. Non-invasive stool-based tests such as the fecal immunochemical test (FIT) are widely implemented but are limited by imperfect sensitivity and specificity [5,6]. These limitations underscore the need for reliable molecular biomarkers that could improve risk stratification and guide personalized management.

Molecular profiling has substantially advanced CRC management. Biomarkers such as microsatellite instability (MSI) and mismatch repair deficiency (dMMR), KRAS/NRAS, BRAF, PIK3CA, and HER2 have well-established prognostic and predictive value, particularly in the context of systemic therapy selection [7,8,9]. In contrast, the clinical utility of tumor suppressor proteins p53 and PTEN remains less clearly defined, despite their central role in tumor biology.

The p53 protein, encoded by the *TP53* gene, is often referred to as the “guardian of the genome.” It regulates DNA repair, cell cycle arrest, senescence, and apoptosis in response to genomic damage. *TP53* alterations are among the most frequent molecular events in CRC, occurring in approximately 40–60% of tumors. Distinct immunohistochemical p53 expression patterns—overexpression, null pattern, and wild-type expression—have demonstrated high concordance with underlying *TP53* mutational status in colorectal carcinoma [10,11,12].

PTEN is a dual-specificity phosphatase that negatively regulates the PI3K/AKT/mTOR signaling pathway, acting as a critical inhibitor of tumor cell proliferation, survival, and angiogenesis. Loss of PTEN function in CRC may result from genetic or epigenetic alterations, including mutations, deletions, promoter hypermethylation, and post-transcriptional regulation. Notably, PTEN dysregulation can occur in the absence of detectable genomic alterations, reflecting complex regulatory mechanisms such as microRNA-mediated repression and competing endogenous RNA (ceRNA) networks [13,14,15,16,17].

Given their central roles in tumor biology, p53 and PTEN represent biologically plausible prognostic biomarkers in CRC. However, their clinical relevance remains uncertain, with published studies reporting heterogeneous results. Therefore, the present study aimed to evaluate the expression of p53 and PTEN in a well-defined surgical cohort of patients with colorectal adenocarcinoma and to assess their prognostic significance in relation to established clinicopathological factors.

## 2. Materials and Methods

### 2.1. Study Population

This study was designed as a retrospective cohort study of patients with CRC. The study population comprised 103 consecutive patients who underwent surgical resection for primary CRC at a municipal hospital in Bytom, Poland, between April 2017 and July 2019.

Clinical and pathological data were retrieved from institutional medical records and included demographic characteristics (age and sex), histopathological features (tumor grade and TNM classification), and follow-up outcomes. Overall survival (OS) was defined as the time from surgery to death from any cause or last follow-up. Survival data were updated until 1 June 2024. Age at diagnosis was recorded for all patients. Because all patients underwent surgical treatment shortly after diagnostic work-up, age at diagnosis closely corresponded to age at surgery and was used in survival analyses. The date and cause of death were verified using both hospital records and national death registry data. Information regarding previous colonoscopic surveillance, screening history, and hereditary CRC syndromes was not consistently available in the medical records and was therefore not included in the analysis.

Eligible cases required a histologically confirmed diagnosis of colorectal adenocarcinoma and availability of formalin-fixed paraffin-embedded (FFPE) tumor tissue suitable for immunohistochemical analysis. Patients with missing follow-up information were excluded. Complete follow-up data were available for all included patients. However, missing values in selected clinicopathological variables, including TNM classification, reduced the sample size available for multivariate analyses. Therefore, Cox proportional hazards regression models were performed using a complete-case approach.

The study was conducted in accordance with the Declaration of Helsinki and approved by the Institutional Ethics Committee (Resolution No. KNW/0022/KB/4/14).

### 2.2. Immunohistochemical Analysis

Immunohistochemical analysis of PTEN and p53 expression was performed on formalin-fixed paraffin-embedded (FFPE) colorectal carcinoma tissue sections.

Consecutive sections obtained from the same FFPE tissue block were stained separately for p53 and PTEN. Double immunostaining was not performed.

Four-micrometer-thick sections were cut from FFPE tissue blocks and mounted on glass slides. The sections were subsequently deparaffinized in xylene and rehydrated through a graded ethanol series.

Antigen retrieval was performed using heat-induced epitope retrieval. For PTEN staining, slides were heated for 20 min at 98 °C in TRIS/EDTA buffer (10 mmol/L, pH 9.0). For p53 staining, antigen retrieval was carried out in citrate buffer (pH 6.0).

Endogenous peroxidase activity was blocked by incubation with 3% hydrogen peroxide. The sections were then incubated with primary antibodies. PTEN expression was detected using a monoclonal rabbit anti-human PTEN antibody (clone 138G6, Cell Signaling Technology, Danvers, MA, USA), raised against the last 100 C-terminal amino acids of the PTEN protein. The antibody specificity had been previously validated by the manufacturer and in published studies. The antibody was diluted 1:75 in Background Reducing Antibody Diluent (Dako, Glostrup, Denmark) and incubated for 30 min at room temperature.

For p53 detection, a monoclonal antibody against p53 (clone DO-7, Dako, Glostrup, Denmark) was used.

Immunoreactivity was visualized using the EnVision+ System (Dako, Glostrup, Denmark), consisting of a horseradish peroxidase-labeled polymer conjugated to affinity-purified goat anti-rabbit Fab’ antibody fragments. 3,3′-diaminobenzidine (DAB) served as the chromogenic substrate.

Slides were counterstained with hematoxylin and mounted using AquaMount (Dako, Glostrup, Denmark). All slides were evaluated under light microscopy.

For PTEN assessment, slides were independently evaluated by a single experienced pathologist blinded to clinical and survival data. Semi-quantitative evaluation was performed according to the method originally described by Perren et al. (1999) and subsequently applied in colorectal cancer studies [18,19,20].

Representative tumor areas were analyzed at ×200 magnification. PTEN expression in tumor cells was compared with adjacent normal epithelium or stromal cells, which served as an internal positive control. PTEN deficiency was defined as complete absence of cytoplasmic staining or markedly reduced staining intensity compared with adjacent normal epithelial or stromal cells. Tumors demonstrating staining intensity equal to or stronger than that observed in adjacent normal tissue were classified as PTEN-positive. Cell line preparations with known PTEN status served as positive controls, while negative controls were prepared by omitting the primary antibody.

p53 expression was evaluated based on nuclear staining patterns in tumor cells and categorized into overexpression, null, and wild-type patterns, as previously described [21,22,23].

For statistical analyses, overexpression and null patterns were classified as aberrant p53 expression, whereas the wild-type pattern was considered as retained physiological expression.

### 2.3. Surgical Procedure

All patients underwent open surgical resection for primary colorectal adenocarcinoma according to standard oncological principles. Following exploration of the abdominal cavity to assess tumor extent and the presence of metastatic disease, the affected segment of the colon or rectum was resected with adequate proximal and distal margins.

High ligation of the corresponding vascular pedicle and regional lymphadenectomy were performed in accordance with contemporary colorectal cancer surgical standards. Reconstruction was achieved by primary anastomosis whenever feasible; otherwise, a protective or definitive stoma was created.

All procedures were performed with curative intent by experienced colorectal surgeons, aiming to achieve complete (R0) tumor resection.

### 2.4. Statistical Analysis

Statistical analyses were performed using IBM SPSS Statistics. Descriptive statistics were calculated for all variables. The Shapiro–Wilk test was used to assess normality of continuous variables. Although survival time deviated from normality, parametric tests were used for exploratory comparisons only, and the main survival analyses were based on Kaplan–Meier estimation and Cox proportional hazards regression. One-way and two-way between-subjects analyses of variance (ANOVA) were used as exploratory analyses of observed survival times across molecular groups defined by p53 expression status and PTEN deficiency. These analyses were exploratory, based on observed survival times without adjustment for censoring, and should be interpreted with caution.

The cohort included 36 patients with wild-type p53 expression and retained PTEN expression, 26 patients with wild-type p53 expression and PTEN deficiency, 8 patients with aberrant p53 expression and retained PTEN expression, and 31 patients with concurrent aberrant p53 expression and PTEN deficiency.

Overall survival (OS) was defined as the time from surgery to death from any cause or last follow-up. Survival distributions were estimated using the Kaplan–Meier method and visually compared between groups. To assess the independent prognostic value of p53 expression status and PTEN status, multivariate Cox proportional hazards regression models were fitted including p53 expression status, PTEN status, T, N, and M classification, histological grade, age, and sex. Due to missing data in selected clinicopathological variables, Cox regression analyses were conducted using a complete-case approach, resulting in an analyzable cohort of 51 patients. Subgroup analyses were performed separately according to p53 expression status and PTEN status. The proportional hazards assumption was assessed graphically. The level of statistical significance was set at α = 0.05.

## 3. Results

The study cohort comprised 103 patients with colorectal adenocarcinoma. The mean age at diagnosis was 66.6 ± 10.2 years. The distribution of sex was nearly equal, with 52 males and 51 females.

The mean overall survival time was 1518.2 ± 804.7 days. During the follow-up period, 43 patients (41.7%) died. Detailed baseline characteristics of the study population are summarized in Table 1.

Descriptive statistics for overall survival are presented in Table 2. The mean survival time was 1518.2. ± 804.7 days, with a median of 1832 days. The distribution of survival time deviated from normality (Shapiro–Wilk test, *p* < 0.001), although skewness (−0.52) and kurtosis (−1.12) remained within acceptable limits.

A two-way between-subjects analysis of variance (ANOVA) was conducted as an exploratory analysis to evaluate the effects of aberrant p53 expression and PTEN deficiency on observed overall survival time in the full cohort (n = 103). Descriptive statistics are presented in Table 3.

**Figure 1:**
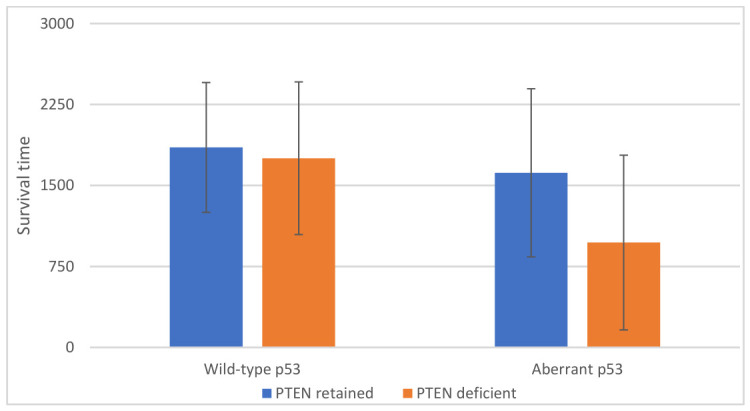
Mean observed overall survival time according to p53 expression status and PTEN deficiency. Error bars represent standard deviations.

Simple effects analyses showed that PTEN deficiency significantly reduced survival only in tumors with aberrant p53 expression (F(1, 99) = 7.39; *p* = 0.008), whereas the effect of aberrant p53 expression was significant only in PTEN-deficient tumors (F(1, 99) = 11.47; *p* = 0.001). The results are visualized in Figure 1.

A one-way between-subjects analysis of variance (ANOVA) was conducted to compare all four patient groups. A statistically significant effect was observed, F(3, 54.87) = 8.83; *p* < 0.001. Post-hoc analyses using the Dunnett test revealed two statistically significant differences. The shortest survival time was found in the group with both aberrant p53 expression and PTEN deficiency, compared with patients without either alteration (*p* < 0.001) and those with wild-type p53 expression and PTEN deficiency (*p* = 0.018). This group also showed a trend toward significance when compared with patients with aberrant p53 expression and retained PTEN expression (*p* = 0.099). The remaining comparisons were not statistically significant.

Because the above analyses were based on observed survival time without accounting for censoring, survival distributions were subsequently evaluated using the Kaplan–Meier methodology.

**Figure 2:**
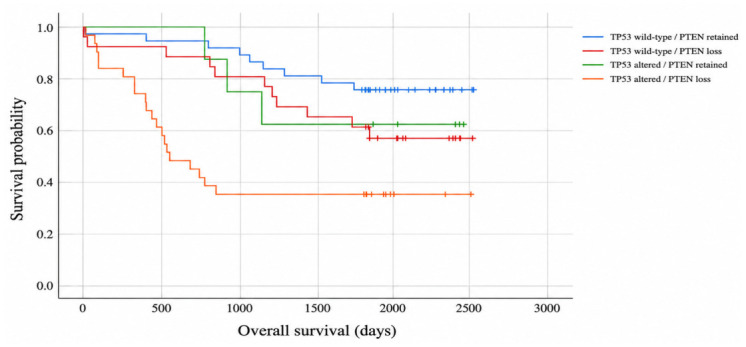
Kaplan–Meier estimates of overall survival according to p53 expression status and PTEN status.

Overall survival was analyzed according to p53 expression status and PTEN status. The shortest survival time was observed in patients harboring both aberrant p53 expression and PTEN deficiency, whereas the longest survival was noted in patients without either alteration. Kaplan–Meier analysis demonstrated statistically significant differences in overall survival among the molecular subgroups. Kaplan–Meier curves are presented in Figure 2.

To determine whether aberrant p53 expression and PTEN deficiency represent independent prognostic factors beyond established clinicopathological variables, multivariate Cox proportional hazards regression was performed including p53 expression status, PTEN status, T, N, and M classification, histological grade, age, and sex. The number of events per variable was limited; therefore, the results of multivariate analyses should be interpreted with caution.

The overall model was statistically significant (χ^2^(9) = 50.81; *p* < 0.001). In the multivariate model, advanced nodal status (N stage; HR = 4.62; *p* = 0.01) and increasing age (HR = 1.11 per year; *p* = 0.003) were independently associated with shorter overall survival. Histological grade (*p* = 0.096) and male sex (*p* = 0.055) showed trends toward statistical significance.

p53 expression status (*p* = 0.527) and PTEN status (*p* = 0.507) were not independently associated with overall survival after adjustment for clinicopathological variables. These findings suggest that the prognostic impact of aberrant p53 expression and PTEN deficiency observed in unadjusted analyses may reflect their association with established clinicopathological factors. Detailed results are presented in Table 4.

Exploratory subgroup analyses were performed stratified according to p53 expression status and PTEN status. In the p53 wild-type subgroup, the multivariate model was statistically significant (χ^2^(8) = 22.27; *p* = 0.004). Increasing age remained the only independent predictor of shorter overall survival (HR = 1.28; *p* = 0.008). Detailed results are presented in Table 5. In the subgroup with aberrant p53 expression, the model could not be reliably fitted due to instability of estimates, likely related to the limited number of events. These analyses should be considered exploratory and hypothesis-generating only. The corresponding results are shown in Table 6.

In the subgroup with retained PTEN expression, the multivariate model was statistically significant (χ^2^(8) = 32.31; *p* < 0.001). Advanced T stage (*p* = 0.021), higher N stage (*p* = 0.025), and increasing age (*p* = 0.045) were independently associated with shorter overall survival. Further details are provided in Table 7.

In the PTEN-deficient subgroup, the multivariate model demonstrated instability of parameter estimates and should be considered exploratory and hypothesis-generating only. Given the limited sample sizes in subgroup analyses, these findings should be interpreted with caution. The results are summarized in Table 8.

No statistically significant associations were observed between PTEN status and clinicopathological variables. Similarly, no statistically significant associations were found between p53 expression status and clinicopathological parameters. However, trends toward association were observed between aberrant p53 expression and more advanced N stage (*p* = 0.064) and M stage (*p* = 0.051). Detailed results are presented in Table 9. The strength of these associations, measured by Cramér’s V coefficient, was low (V = 0.29 and V = 0.26, respectively).

Age did not differ significantly between molecular subgroups defined by p53 expression status and PTEN status (Kruskal–Wallis test, *p* = 0.959). The corresponding results are shown in Table 10.

No significant differences in age were observed between the molecular subgroups, suggesting that age distribution did not differ across molecular subgroups defined by p53 expression status and PTEN status.

## 4. Discussion

The present study examined the prognostic relevance of aberrant p53 expression (as an immunohistochemical surrogate of TP53 alteration) and PTEN deficiency in a retrospective surgical cohort of colorectal adenocarcinoma. In unadjusted analyses based on observed survival and Kaplan–Meier estimates, both biomarkers were associated with shorter overall survival, and tumors harboring concurrent aberrant p53 expression and PTEN deficiency consistently represented the subgroup with the poorest outcomes. However, in multivariate Cox proportional hazards models adjusted for established clinicopathological variables, nodal status and age remained the only independent predictors of survival, whereas p53 expression status and PTEN status did not retain independent prognostic significance.

These findings underscore the distinction between biological association and independent clinical prognostic value and suggest that p53 and PTEN reflect underlying tumor biology rather than serving as independent prognostic biomarkers. Although alterations involving p53 and PTEN signaling pathways mark biologically aggressive disease, their adverse survival effect appears to be largely mediated through established determinants of tumor progression, particularly lymph node involvement.

### 4.1. TP53 Alterations in Colorectal Cancer

*TP53* is a central tumor suppressor regulating DNA repair, cell-cycle arrest, apoptosis, and senescence. Mutations frequently result in both loss-of-function and gain-of-function properties that promote genomic instability and invasion [24]. In colorectal cancer (CRC), TP53 alterations are among the most common molecular events and have been associated with adverse outcomes in multiple cohorts and pooled analyses [25,26,27].

In the present cohort, aberrant p53 expression was observed in a substantial proportion of tumors, consistent with previous studies reporting TP*53* alterations in approximately 40–60% of colorectal cancers, and was associated with shorter overall survival in unadjusted analyses, in line with previous reports linking TP53 abnormalities to impaired apoptosis and therapeutic resistance [25,26]. However, the independent prognostic value of aberrant p53 expression remains heterogeneous across studies and appears context-dependent [27,28]. In particular, its effect may differ between microsatellite-stable (MSS) and MSI-high tumors, reflecting distinct evolutionary trajectories and immune microenvironments [29,30]. This molecular heterogeneity likely contributes to the attenuation of p53 significance after multivariate adjustment in our model.

### 4.2. PTEN Deficiency and Pathway Dysregulation

PTEN is a key negative regulator of the PI3K/AKT/mTOR pathway and plays a critical role in maintaining genomic integrity and controlling cell survival [31]. PTEN loss has been associated with advanced stage, lymph node involvement, and worse survival in CRC [13,32]. In our cohort, PTEN deficiency was detected in more than half of tumors and was associated with shortened survival in unadjusted analyses.

Importantly, large-scale genomic studies suggest that the prognostic implications of *PTEN* alterations depend on molecular context, including microsatellite status and co-occurring alterations involving *TP53* [33]. Our findings are consistent with this concept: the adverse prognostic impact of PTEN deficiency appeared to be conditioned by p53 expression status rather than uniformly distributed across all tumors.

### 4.3. Combined Disruption of TP53 and PTEN Pathways

The most pronounced survival disadvantage was observed in tumors harboring concurrent aberrant p53 expression and PTEN deficiency. Although the interaction term in the two-way ANOVA did not reach statistical significance, simple-effects analyses demonstrated that PTEN deficiency significantly reduced survival only in the subgroup with aberrant p53 expression. Patients with disruption of both pathways had the shortest median overall survival.

This observation is biologically coherent. PTEN and p53 signaling intersect at multiple regulatory levels: PTEN can enhance p53 stability by modulating MDM2 activity, while p53 can transcriptionally upregulate *PTEN*, forming a tumor-suppressive feedback loop [31]. Simultaneous disruption of both axes is therefore expected to produce compounded impairment of genomic surveillance and apoptosis, resulting in a more aggressive phenotype. The combined disruption of *TP53* and *PTEN* signaling may therefore represent a biologically meaningful marker of aggressive tumor behavior rather than an independent prognostic factor when classical clinicopathological variables are considered.

**Figure 3:**
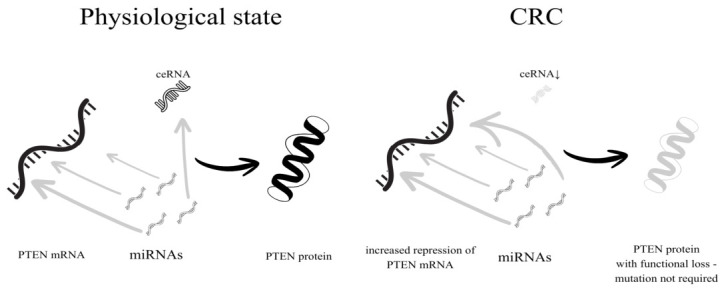
ceRNA-mediated regulation as a mechanism of functional PTEN loss in colorectal cancer. In physiological conditions, competing endogenous RNAs (ceRNAs) sequester microRNAs and limit repression of PTEN mRNA, preserving PTEN protein expression. In colorectal cancer, reduced ceRNA buffering shifts microRNA activity toward PTEN transcripts, resulting in decreased PTEN protein levels and downstream pathway activation, even in the absence of genomic mutation.

As illustrated schematically in Figure 3, PTEN deficiency does not necessarily require genomic mutation. According to the competing endogenous RNA (ceRNA) hypothesis, non-coding transcripts sharing microRNA response elements with *PTEN* (e.g., *PTENP1* or CRC-related lncRNAs) can modulate microRNA availability and thereby regulate PTEN translation [16,34]. Reduced ceRNA buffering may increase microRNA-mediated repression of PTEN mRNA, leading to functional protein loss even in the absence of DNA-level alteration. This mechanistic framework provides a plausible explanation for why immunohistochemically detected PTEN deficiency may capture clinically relevant pathway disruption not fully reflected by genomic assays alone [16,34]. It also supports the biological plausibility of the observed interaction between PTEN deficiency and aberrant p53 expression.

The particularly unfavorable prognosis observed in tumors with concurrent loss of PTEN and aberrant p53 expression may also be interpreted in the context of the concept of obligate haploinsufficiency proposed by Pier Paolo Pandolfi. This model suggests that even a partial reduction in the dosage or activity of tumor suppressor genes can significantly impair their biological function and promote tumor progression. Simultaneous disruption of *PTEN* and p53 signaling may therefore result in a synergistic weakening of tumor suppressive mechanisms, affecting both genomic stability and regulation of the PI3K/AKT pathway. Such combined impairment of two major tumor suppressor pathways may contribute to the more aggressive clinical behavior observed in these tumors [35].

### 4.4. Multivariate Adjustment and Clinical Interpretation

Despite the clear survival gradients observed in unadjusted analyses, multivariate Cox regression identified advanced nodal status and age as the only independent predictors of overall survival. The loss of independent significance for p53 expression status and PTEN status after adjustment suggests that their prognostic impact is closely intertwined with tumor stage and progression dynamics.

These findings align with prior reports indicating that molecular markers may complement—but do not necessarily outperform—classical clinicopathological parameters in CRC prognosis [13,27]. Thus, alterations involving p53 and PTEN signaling pathways appear to refine biological stratification rather than replace established staging systems.

Subgroup Cox analyses demonstrated model instability in selected subsets, particularly those with small event numbers, and should therefore be interpreted as exploratory. Nonetheless, the overall pattern supports a context-dependent model in which the prognostic relevance of PTEN deficiency is amplified in the presence of aberrant p53 expression.

### 4.5. Study Limitations

First, the retrospective design and single-center setting may have introduced selection bias, thereby limiting the generalizability of the findings. Although the cohort size allowed detection of statistically significant associations in unadjusted analyses, the number of cases was moderate, and subgroup analyses—particularly in the multivariate and stratified models—were underpowered. In addition, the absence of an independent validation cohort restricts the robustness of the conclusions. Missing clinicopathological data resulted in a reduced sample size for complete-case multivariate Cox regression, which may have influenced model stability.

Second, assessment of p53 expression and PTEN status was based exclusively on immunohistochemistry performed on archival FFPE material. The dichotomous classification of p53 expression into “mutation-associated” and “wild-type” patterns and PTEN into “deficient” and “retained” expression simplified a biologically continuous spectrum and may have masked intermediate phenotypes. Although standardized scoring criteria were applied and evaluation was performed by an experienced pathologist blinded to outcomes, inter-observer variability cannot be entirely excluded. Integration of complementary molecular analyses such as targeted sequencing of *TP53* and *PTEN*, copy-number assessment, or methylation profiling together with IHC-based protein expression assessment would provide stronger mechanistic validation.

Third, comprehensive molecular stratification was not available. Data regarding microsatellite instability (MSI) status and KRAS/NRAS, BRAF, or PIK3CA mutations, as well as detailed adjuvant treatment information, were not incorporated into the analysis. Given the well-established heterogeneity of colorectal cancer, the absence of these variables limited the ability to perform integrated molecular modeling and to explore interactions between tumor suppressor pathways and established oncogenic drivers. Similarly, certain clinical factors—such as comorbidities, precise tumor location, and postoperative complications—were not included and may have influenced survival outcomes.

Detailed information regarding previous colonoscopic surveillance, screening history, hereditary CRC syndromes, and molecular assessment of preoperative biopsy specimens was unavailable or incomplete in this retrospective cohort. Therefore, the impact of screening or surveillance on survival outcomes according to PTEN and p53 expression status could not be reliably assessed. Similarly, paired analysis of preoperative biopsy specimens and resection samples was not performed; therefore, temporal changes in PTEN and p53 expression during tumor progression could not be evaluated.

Despite these limitations, the present study provides clinically relevant evidence that concurrent disruption of p53 and PTEN signaling identifies a biologically aggressive subset of colorectal cancers. The findings support further prospective, multi-center investigations incorporating integrated molecular profiling to clarify the context-dependent prognostic impact of combined disruption of TP53 and PTEN signaling pathways.

## 5. Conclusions

The present study demonstrated that aberrant p53 expression and PTEN deficiency were associated with shorter overall survival in patients with colorectal cancer in unadjusted analyses. The coexistence of both molecular alterations defined a subgroup with particularly poor prognosis, supporting a biologically plausible interaction between the TP53 and PTEN tumor suppressor pathways.

However, after adjustment for established clinicopathological variables in multivariate Cox regression analysis, nodal status and age remained the only independent predictors of survival, whereas p53 expression status and PTEN status did not retain independent prognostic significance.

These findings suggest that aberrant p53 expression and PTEN deficiency may reflect an underlying aggressive tumor phenotype rather than function as independent prognostic markers. Integrated assessment of disruption of the TP53 and PTEN signaling pathways may contribute to biological risk stratification; however, its independent clinical utility requires validation in larger, prospectively characterized cohorts incorporating comprehensive molecular profiling.

## Figures and Tables

**Table 1:** Baseline characteristics of the study group.

Variable	Value
Number of patients	103
Age [years]	66.6 ± 10.2
Sex, n (Male/Female)	52/51
Overall survival time [days, mean ± SD]	1518.2 ± 804.7
Deaths (n, %)	43 (41.7%)

Note: Data are presented as mean ± standard deviation (SD) or number of patients (n).

**Table 2:** Descriptive statistics for survival time.

	M	Me	SD	Sk.	Kurt.	Min.	Max.	W	*p*
Survival time	1518.17	1832	804.68	−0.52	−1.12	3	2529	0.89	<0.001

Note: M—mean; Me—median; SD—standard deviation; Sk.—skewness; Kurt.—kurtosis; Min and Max—minimum and maximum values; W—Shapiro–Wilk statistic; *p*—significance level.

**Table 3:** Observed overall survival according to p53 expression status and PTEN deficiency.

p53 Expression Status	PTEN Deficiency	M	SD
Wild-type	No	1852.97	601.28
	Yes	1617.41	778.68
	Overall	1753.59	685.99
Aberrant	No	1752.63	706.98
	Yes	971.61	809.40
	Overall	1131.82	843.42
Overall	No	1835.13	613.87
	Yes	1272.24	852.62

Note: M—mean; SD—standard deviation. A significant main effect of PTEN deficiency was observed (F(1, 99) = 8.90; *p* = 0.004; η^2^ = 0.08), with longer observed survival in patients without PTEN deficiency. A significant main effect of aberrant p53 expression was also found (F(1, 99) = 4.80; *p* = 0.031; η^2^ = 0.05). The interaction between p53 expression pattern and PTEN deficiency was not statistically significant (F(1, 99) = 2.56; *p* = 0.113; η^2^ = 0.03).

**Table 4:** Multivariate Cox proportional hazards regression analysis for overall survival.

	B	SE	Wald	*p*	Exp(B)
P53	0.45	0.70	0.40	0.527	1.56
PTEN	0.47	0.70	0.44	0.507	1.59
T stage	−1.23	0.78	2.48	0.116	0.29
N stage	1.53	0.59	6.70	0.01	4.62
M stage	0.19	1.09	0.03	0.86	1.21
Grade	0.88	0.53	2.77	0.096	2.42
Age	0.10	0.03	9.12	0.003	1.11
Sex	1.20	0.62	3.70	0.055	3.31

B—regression coefficient; SE—standard error; Wald—Wald χ^2^ statistic; *p*—significance level; Exp(B)—hazard ratio (HR). HR > 1 indicates increased risk of death.

**Table 5:** Multivariate Cox regression analysis in the p53 wild-type subgroup.

	B	SE	Wald	*p*	Exp(B)
PTEN	−0.68	1.08	0.39	0.532	0.51
T stage	−2.30	1.26	3.31	0.069	0.10
N stage	1.22	1.00	1.47	0.225	3.38
M stage	−1.42	2.44	0.34	0.563	0.24
Grade	−0.27	1.01	0.07	0.790	0.76
Age	0.24	0.09	7.00	0.008	1.28
Sex	1.41	1.21	1.37	0.241	4.11

B—regression coefficient; SE—standard error; Wald—Wald χ^2^ statistic; *p*—significance level; Exp(B)—hazard ratio (HR). HR > 1 indicates increased risk of death.

**Table 6:** Exploratory multivariate Cox regression analysis in the subgroup with aberrant p53 expression.

	B	SE	Wald	*p*	Exp(B)
PTEN	13.10	276.09	0.00	0.962	486,897.31
T stage	−1.72	1.92	0.80	0.371	0.18
N stage	0.45	1.43	0.10	0.750	1.58
M stage	−0.40	1.67	0.06	0.810	0.67
Grade	0.88	0.91	0.94	0.331	2.41
Age	0.01	0.06	0.03	0.869	1.01
Sex	−0.39	0.85	0.21	0.646	0.68

B—regression coefficient; SE—standard error; Wald—Wald *χ^2^* statistic; *p*—significance level; Exp(B)—hazard ratio (HR). HR > 1 indicates increased risk of death. *The model demonstrated substantial instability of parameter estimates and should be interpreted with caution.*

**Table 7:** Multivariate Cox regression analysis in the subgroup with retained PTEN expression.

	B	SE	Wald	*p*	Exp(B)
T stage	−2.20	0.95	5.30	0.021	0.11
N stage	1.87	0.83	5.05	0.025	6.50
M stage	1.05	1.16	0.83	0.362	2.87
Grade	0.69	0.59	1.35	0.245	1.99
Age	0.07	0.04	4.02	0.045	1.07
Sex	1.07	0.69	2.43	0.119	2.92
p53 expression status	0.76	0.88	0.75	0.386	2.14

B—regression coefficient; SE—standard error; Wald—Wald χ^2^ statistic; *p*—significance level; Exp(B)—hazard ratio (HR). HR > 1 indicates increased risk of death.

**Table 8:** Exploratory multivariate Cox regression analysis in the PTEN-deficient subgroup.

	B	SE	Wald	*p*	Exp(B)
T stage	−2.83	13.92	0.04	0.839	0.06
N stage	12.29	26.60	0.21	0.644	217,536.97
M stage	13.41	766.34	0.00	0.986	666,078.90
Grade	6.16	16.22	0.14	0.704	471.00
Age	2.28	3.91	0.34	0.560	9.76
Sex	17.80	25.71	0.48	0.489	53,625,655.26
p53 expression status	−5.33	96.43	0.00	0.956	0.01

B—regression coefficient; SE—standard error; Wald—Wald χ^2^ statistic; *p*—significance level; Exp(B)—hazard ratio (HR). HR > 1 indicates increased risk of death.

**Table 9:** Association between p53 expression pattern and clinicopathological variables.

		p53 Expression Status				
		Wild-Type		Aberrant		Exact Fisher Test
Variable		N	%	N	%	*p*
T stage	1	3	5.0%	1	2.7%	0.638
	2	15	25.0%	14	37.8%	
	3	37	61.7%	20	54.1%	
	4	5	8.3%	2	5.4%	
N stage	0	30	50.8%	10	27.0%	0.064
	1	13	22.0%	7	18.9%	
	2	9	15.3%	13	35.1%	
	3	5	8.5%	6	16.2%	
M stage	0	53	91.4%	26	72.2%	0.051
	1	4	6.9%	7	19.4%	
	2	1	1.7%	3	8.3%	
Grade	0	5	12.5%	5	20.0%	0.735
	1	2	5.0%	2	8.0%	
	2	22	55.0%	13	52.0%	
	3	11	27.5%	5	20.0%	

**Table 10:** Age distribution across p53/PTEN molecular subgroups.

	Wild-Type p53/ PTEN Retained (n = 36)		Wild-Type p53/ PTEN Deficient (n = 26)		Aberrant p53/ PTEN Retained (n = 8)		Aberrant p53/ PTEN Deficient (n = 31)			
	M	SD	M	SD	M	SD	M	SD	H	*p*
Age	66.39	8.73	66.85	10.02	66.38	12.98	66.81	10.08	0.08	0.959

Age is presented as mean (M) and standard deviation (SD). Differences between groups were assessed using the Kruskal–Wallis test.

## Data Availability

The raw data supporting the findings of this study are available from the corresponding author upon reasonable request. The data are not publicly available because they contain potentially identifiable clinical information obtained from hospital medical records, and their sharing is restricted by patient confidentiality requirements and institutional ethical regulations.
